# Encapsulation Strategies for Lemon Essential Oil in Lipid-Based Food Systems: Recent Advances and Applications in Oxidative Stability

**DOI:** 10.3390/foods15050950

**Published:** 2026-03-07

**Authors:** Louiza Himed, Salah Merniz, Rofia Djerri, Belkis Akachat, Hadria Boussioud, Asmaa Berkati, Maria D’Elia, Luca Rastrelli

**Affiliations:** 1 Laboratory of Biotechnology and Food Quality (BIOQUAL), Institute of Nutrition, Food and Agro-Food Technologies (INATAA), University of Constantine 1, Constantine 25000, Algeria; 2 Institute of Industrial Hygiene and Safety, University Batna 2, Batna 05000, Algeria; 3 Laboratoire de Biomathématiques, Biochimie, Biophysique et Scientométrie (L3BS), Faculté des Sciences de la Nature Et de la Vie, Université de Bejaia, Bejaia 06000, Algeria; 4 National Biodiversity Future Center (NBFC), 90133 Palermo, Italy; mdelia@unisa.it; 5 Department of Pharmacy, University of Salerno, Via Giovanni Paolo II, 132, 84084 Salerno, Italy; 6 Dipartimento di Scienze della Terra e del Mare, University of Palermo, 90123 Palermo, Italy

**Keywords:** natural preservatives, clean-label ingredients, functional lipids, sensory quality preservation, fat-containing foods, industrial food formulation

## Abstract

Essential oils, particularly lemon essential oil (LEO), have attracted increasing interest as natural antimicrobial and antioxidant agents for food preservation. However, their direct incorporation into food systems is limited by high volatility, poor water solubility, oxidative instability, and potential sensory impacts. Encapsulation has emerged as an effective technological strategy to overcome these constraints by improving the stability and controlled release of LEO, especially in lipid-based food matrices such as margarine. This review critically summarizes recent advances (2020–2024) in the extraction, physicochemical characterization, and encapsulation of LEO, with particular emphasis on food-grade delivery systems, including biopolymers and inorganic carriers such as silica. Encapsulation efficiency, protection mechanisms, and release behavior are discussed in relation to oxidative stability and functional performance in real food applications. Special attention is devoted to light margarine as a model lipid system, highlighting the advantages and limitations of different encapsulation strategies in delaying lipid oxidation while preserving sensory quality. Finally, emerging challenges related to scalability, regulatory acceptance, and safety, together with future perspectives on smart food packaging and sustainable encapsulation technologies, are outlined to support the effective translation of LEO-based systems into industrial food applications.

## 1. Introduction

In recent years, the global food industry has undergone a marked shift toward the use of natural preservatives, driven by increasing consumer demand for clean-label, additive-free products capable of maintaining quality and safety throughout extended shelf life. This transition aligns with broader objectives related to sustainable food systems and public health, encouraging manufacturers to replace synthetic additives, often associated with potential health concerns, with plant-derived alternatives [1,2].

Among the most promising natural compounds are essential oils (EOs), volatile aromatic substances extracted from various plant organs, known for their pronounced antimicrobial, antifungal, and antioxidant activities. Lemon essential oil (LEO), obtained from the peel of *Citrus limon*, is particularly noteworthy due to its high content of monoterpenes, especially limonene (up to 70%), along with β-pinene, γ-terpinene, citral, and linalool [3]. These constituents are responsible not only for the characteristic citrus aroma but also for the biological activity of LEO. Numerous studies have demonstrated its ability to inhibit lipid oxidation and suppress the growth of major foodborne pathogens, including *Escherichia coli*, *Listeria monocytogenes*, and *Salmonella* spp. [4,5,6].

Despite these advantages, the direct incorporation of LEO into food matrices presents significant technological challenges. Its high volatility, low water solubility, and chemical instability in the presence of heat, oxygen, or light can lead to rapid degradation of active compounds and inconsistent functional performance [7]. In addition, the intense citrus aroma of LEO may adversely affect the sensory properties of certain food products when applied in free form [8].

To overcome these limitations, encapsulation technologies have emerged as an effective strategy for protecting, stabilizing, and controlling the release of LEO in food systems. Encapsulation can be defined as an entrapment technique in which a compound is enclosed within a more stable physical barrier, remaining localized at the core of a protective structure. The encapsulated substance is commonly referred to as the core, active phase, or payload, whereas the encapsulating material is typically known as the matrix, membrane, wall, coating, or capsule [9]. Encapsulation involves immobilizing essential oil droplets within carrier matrices such as biopolymers, silica particles, or lipid-based materials, thereby shielding the active compounds from environmental stressors and improving their compatibility with food environments [10]. Recent advances in nanoencapsulation, including antisolvent dialysis, as well as complex coacervation, spray-drying, and sol–gel techniques, have enabled improved retention of bioactivity, mitigation of sensory impact, and enhanced antimicrobial and antioxidant performance [11]. Among emerging encapsulation techniques, electrospraying has attracted increasing attention for the encapsulation of bioactive compounds such as essential oils, due to its ability to produce fine particles and high encapsulation efficiencies. However, its application in food systems remains limited by the need for sophisticated equipment, high energy input, and lower industrial scalability compared to more established techniques such as spray drying or emulsification-based approaches.

For example, Meng et al. [12] developed LEO nanoemulsions capable of effectively inhibiting fungal spoilage on kiwifruits, demonstrating high physicochemical stability and biological efficacy. Similarly, Zaki et al. [13] reported that LEO-loaded nanoliposomes enhanced bioavailability and persistence in complex food matrices such as soups. More recently, Salah et al. [14] showed that the incorporation of encapsulated LEO into light margarine significantly delayed lipid oxidation while preserving acceptable sensory profiles. While several encapsulation systems are traditionally developed in aqueous or multiphase environments, their adaptation to lipid-based food matrices requires a critical evaluation of carrier–oil interactions, oxygen permeability, and release kinetics. For this reason, light margarine is discussed here as a representative lipid system to contextualize and compare encapsulation strategies under realistic oxidative conditions

Against this background, the present review aims to synthesize advances published between 2020 and 2024 on LEO encapsulation, with particular focus on:

- the types of encapsulating materials employed (biopolymers, silica, lipid matrices),
- their physicochemical interactions with LEO,
- and the technological implications for food formulation, especially in lipid-based products such as light margarine.

The incorporation of encapsulated LEO into margarine represents a promising strategy to simultaneously enhance oxidative stability, preserve product quality, and meet consumer demand for natural and healthier foods. Accordingly, this review provides a critical assessment of current encapsulation strategies for LEO, highlighting both their advantages and limitations, and outlines future perspectives for natural food preservation and intelligent packaging applications.

## 2. Methodological Approach of the Review

This review is presented as a narrative and critical review aimed at summarizing recent advances in the encapsulation of lemon essential oil (LEO) for food applications. The literature survey focused on peer-reviewed studies published between 2020 and 2024 and indexed in major scientific databases, including Scopus, Web of Science, and PubMed.

The selection of literature prioritized studies addressing food-grade encapsulation materials and technologies, physicochemical characterization of LEO and encapsulated systems, and evaluation of antioxidant or antimicrobial performance in model or real food matrices. Particular attention was given to lipid-based food systems, with light margarine considered a representative model due to its high susceptibility to oxidative degradation.

Rather than adopting a systematic review approach, the present work provides an integrative and application-oriented analysis, highlighting technological trends, key advantages, limitations, and knowledge gaps relevant to the development and industrial translation of LEO-based encapsulation strategies.

## 3. Chemical Composition and Biological Activity of Lemon Essential Oil

Lemon essential oil (LEO), primarily extracted from the peel of *Citrus limon*, is a complex mixture of volatile organic compounds, predominantly monoterpenes, which confer its distinctive citrus aroma and broad spectrum of biological activities. Limonene is the major constituent, typically representing 65–70% of the total oil, serving as a key marker of both quality and bioactivity. Other major components include β-pinene, γ-terpinene, citral (neral and geranial), linalool, and α-terpineol, as well as minor constituents such as aldehydes, ketones, and esters [15].

The biological efficacy of LEO is closely linked to this rich monoterpenic profile. Monoterpenes have been widely reported to exhibit strong antioxidant properties, mainly through their capacity to scavenge reactive oxygen species (ROS), donate hydrogen atoms to neutralize free radicals, and inhibit lipid peroxidation. These effects are particularly relevant in unsaturated fatty acid-rich matrices such as margarine and edible oils. In vitro studies have shown that limonene and γ-terpinene possess marked radical-quenching activity, often comparable to that of synthetic antioxidants such as butylated hydroxytoluene (BHT) and α-tocopherol [16] (Table 1).

In addition to its antioxidant capacity, LEO displays significant antimicrobial activity against a broad range of Gram-positive and Gram-negative microorganisms, including *Listeria monocytogenes*, *Escherichia coli*, *Staphylococcus aureus*, and several fungal species. The antimicrobial mechanism is generally attributed to disruption of microbial cell membranes, increased membrane permeability, and interference with intracellular enzymes and genetic material. Importantly, synergistic interactions among LEO constituents—such as citral, linalool, and limonene—have been reported to enhance antimicrobial efficacy even at low concentrations, likely through cooperative membrane destabilization and increased cytotoxicity toward pathogens [16].

However, the chemical composition of LEO is inherently variable and influenced by multiple biological, agronomic, and technological factors, including:

- cultivar and genetic background of the plant,
- geographical origin and climatic conditions (e.g., sunlight exposure, temperature, soil characteristics),
- fruit maturity stage and harvest timing,
- extraction method (e.g., steam distillation, cold pressing, microwave-assisted hydrodistillation),
- and post-harvest storage conditions, particularly exposure to light, oxygen, and temperature.

Such variability may result in the occurrence of different chemotypes within the same species, leading to substantial differences in both biological efficacy and sensory attributes. For instance, limonene-rich chemotypes are generally associated with enhanced antioxidant performance, whereas citral-dominant profiles may exhibit stronger antimicrobial activity.

Given this compositional variability, standardization and detailed chemical profiling of LEO are essential prerequisites for its reliable application in food systems. Advanced analytical techniques, including gas chromatography–mass spectrometry (GC–MS), Fourier-transform infrared spectroscopy (FTIR), and nuclear magnetic resonance (NMR), are routinely employed to characterize chemical composition, ensure quality control, and guide formulation strategies. Comprehensive profiling enables reproducible bioactivity and supports the rational selection of appropriate encapsulation systems based on the physicochemical properties of the active constituents.

Ultimately, the effectiveness of LEO in food preservation is intimately linked to its precise chemical composition. Consequently, chemometric approaches combined with bioactivity-guided fractionation are increasingly applied to correlate compositional features with functional performance, facilitating the development of standardized, consistent, and effective LEO-based food additives and smart packaging agents.

## 4. Extraction and Characterization Techniques

The extraction of lemon essential oil (LEO) from *Citrus limon* peel represents a critical step that directly influences its chemical composition, volatility, bioactivity, and overall suitability for food applications. The efficiency and selectivity of extraction processes strongly affect the concentration of key bioactive constituents, particularly monoterpenes and oxygenated compounds, which are often thermolabile. In recent years, both conventional and innovative green extraction techniques have been extensively investigated to maximize yield, enhance purity, and preserve the functional integrity of bioactive molecules.

### 4.1. Conventional Extraction Methods

The two most widely applied traditional techniques for LEO extraction at both laboratory and industrial scales are steam distillation (SD) and cold pressing (CP).

Steam distillation (SD) involves passing saturated steam through shredded lemon peels to volatilize essential oil components, which are subsequently condensed and separated into oil and aqueous phases. Although SD is scalable and economically feasible, it may induce thermal degradation, hydrolysis, or isomerization of thermosensitive compounds such as citral and limonene, potentially altering both aroma and biological activity [25].

Cold pressing (CP), also referred to as mechanical expression, relies on the physical rupture of oil glands located in the flavedo (outer peel) to release essential oil without external heat input. This method is particularly suitable for preserving highly volatile and thermolabile compounds, thereby maintaining the authentic aroma profile of fresh citrus fruits. However, CP typically yields lower concentrations of oxygenated terpenes and may result in oils containing higher levels of waxes and pigment residues [26].

### 4.2. Innovative Extraction Techniques

To overcome the limitations associated with conventional methods, several eco-friendly and technology-driven extraction approaches have been developed, aiming to improve extraction efficiency, selectivity, energy sustainability, and preservation of functional compounds.

Microwave-assisted hydrodistillation (MAHD) exploits microwave energy to induce rapid internal heating of plant tissues, leading to efficient rupture of oil glands and accelerated volatilization of essential oils. Compared with SD, MAHD significantly reduces extraction time and energy consumption while minimizing thermal degradation. Several studies have reported enhanced monoterpene retention and increased LEO yield using this approach [27].

Ultrasound-assisted extraction (UAE) is based on acoustic cavitation, which generates microbubbles that collapse violently in the extraction medium, causing mechanical disruption of plant cell walls. This non-thermal technique facilitates oil release at relatively low temperatures, preserving bioactive compounds while shortening processing time and reducing solvent usage [28].

Supercritical CO_2_ extraction (SC-CO_2_) employs carbon dioxide under supercritical conditions (typically above 31 °C and 73 bar) as a green solvent. This method allows selective extraction of non-polar compounds such as limonene, yielding oils of high purity with negligible solvent residues. Despite higher capital and operational costs, SC-CO_2_ is attractive due to its tunable selectivity, low thermal stress, and favorable environmental profile [29].

Ohmic heating extraction (OHE) represents a relatively novel approach in which electrical current is applied to heat plant tissues internally through electrical resistance. This ensures uniform temperature distribution, reduced processing times, and limited oxidation, potentially enhancing the preservation of volatile compounds. Preliminary studies suggest that OHE may outperform conventional heating in both extraction efficiency and retention of sensitive terpenes, although its application to citrus essential oils remains at an early developmental stage [30].

### 4.3. Comparative Assessment and Process Optimization

**Figure 1:**
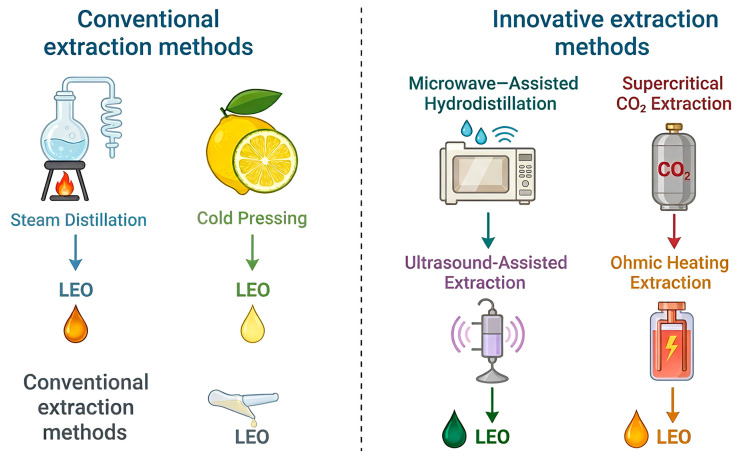
Comparative features of lemon essential oil extraction methods.

Each extraction technique exhibits specific advantages and limitations in terms of selectivity, thermal impact, scalability, and sustainability. Consequently, systematic process optimization, often guided by response surface methodology (RSM) or multi-objective decision-making tools, is essential to tailor extraction parameters such as time, temperature, solvent ratio, and energy input. Such optimization strategies aim to maximize yield while preserving bioactivity-relevant markers. The integration of green extraction principles with analytical quality control is increasingly advocated to ensure reproducibility and consistent quality of LEO intended for food and nutraceutical applications (Figure 1 and Table 2).

### 4.4. Analytical Characterization Techniques

Following extraction, comprehensive chemical characterization of LEO is essential to confirm authenticity, assess bioactive potential, and ensure batch-to-batch consistency. Given the chemical complexity and natural variability of essential oils, the combined use of complementary analytical techniques is required to achieve reliable qualitative and quantitative assessment, particularly for applications in food, pharmaceutical, and cosmetic systems.

Gas chromatography–mass spectrometry (GC–MS) remains the reference technique for qualitative and quantitative profiling of volatile constituents. It enables accurate identification of monoterpenes (e.g., limonene, γ-terpinene) and oxygenated compounds (e.g., citral, linalool) based on retention indices and characteristic mass fragmentation patterns [31]. In addition to compound identification, GC–MS allows chemotype classification and comparison of LEO samples obtained from different cultivars, geographical origins, or extraction methods. Numerous studies have used GC–MS data to correlate chemical profiles with antioxidant and antimicrobial activities, supporting bioactivity-guided selection of LEO for food applications [32].

Fourier-transform infrared spectroscopy (FTIR) is widely applied for rapid fingerprinting and detection of oxidative or thermal degradation of essential oil components. It provides information on functional groups (e.g., C=O, –OH, –C–O–C–) and supports quality control of both free and encapsulated LEO. Several studies have demonstrated that FTIR, particularly when combined with chemometric analysis, is effective for detecting adulteration, monitoring oxidation processes, and assessing chemical stability during storage and processing of citrus essential oils [33].

Nuclear magnetic resonance (NMR) spectroscopy, particularly ^1^H and ^13^C NMR, offers high-resolution structural elucidation and quantitative assessment of key constituents such as citral isomers. Unlike chromatographic techniques, NMR enables direct quantification without prior separation and provides detailed structural information, making it a powerful tool for authenticity verification and detection of adulteration. Although its routine use is limited by cost and technical complexity, recent research increasingly applies NMR for accurate quantification of bioactive compounds in citrus essential oils [34].

Headspace solid-phase microextraction (HS-SPME) coupled with GC or GC–MS provides a solvent-free and highly sensitive approach for analyzing volatile compounds, particularly in emulsified or encapsulated LEO systems. This technique has been extensively employed in research to investigate volatile release kinetics, aroma stability, and migration behavior of LEO in active packaging, edible coatings, and controlled-release formulations, making it especially relevant for real food system applications [35,36].

Collectively, these analytical tools not only support quality assurance and regulatory compliance, but also enable correlation between chemical composition and antioxidant, antimicrobial, or sensory properties, as well as evaluation of LEO stability during processing and storage. Their combined application provides a comprehensive framework for standardization and rational formulation of LEO-based functional ingredients and smart packaging systems.

### 4.5. Chemometric and Multivariate Approaches

The growing complexity of chemical datasets generated by advanced analytical techniques has driven the adoption of multivariate statistical tools for data interpretation. Chemometric approaches such as principal component analysis (PCA), hierarchical cluster analysis (HCA), and partial least squares regression (PLS-R) have become indispensable for the robust evaluation of LEO profiles.

These methods offer several key advantages:

- Chemotype discrimination, allowing classification of LEO samples based on dominant constituents such as limonene, β-pinene, and citral, and differentiation according to geographical origin or extraction method [37,38];
- Assessment of extraction effects, enabling visualization of how techniques such as MAHD and SD influence the relative abundance of key bioactive compounds [39];
- Correlation with bioactivity, whereby coupling PLS-R with antioxidant or antimicrobial data identifies chemical markers most strongly associated with functional performance [40].

Overall, these data-driven approaches enhance quality control, support authenticity verification, and facilitate the development of standardized, high-performance LEO-based formulations for food, cosmetic, and pharmaceutical applications.

## 5. Encapsulation Technologies for Lemon Essential Oil

Encapsulation represents a pivotal technological approach for the stabilization, protection, and controlled delivery of volatile and chemically sensitive compounds such as lemon essential oil (LEO). Owing to its lipophilic nature, high volatility, and susceptibility to oxidative degradation, LEO requires effective protective strategies to preserve its bioactivity during processing, storage, and incorporation into food matrices. Encapsulation technologies play a crucial role in maintaining the functional properties of LEO and enabling its application in food, cosmetic, and pharmaceutical systems [41,42].

### 5.1. Objectives and Functional Benefits of Encapsulation

The direct use of LEO in food systems is limited by rapid volatilization, oxidative instability, and poor dispersibility in aqueous environments. Encapsulation effectively addresses these challenges by providing physical protection against oxidation, heat, and ultraviolet radiation, thereby preserving aromatic and bioactive compounds [43]. In addition, encapsulation enables controlled release during storage, processing, or digestion, allowing sustained bioactivity and moderated sensory impact. Improved dispersibility and compatibility in hydrophilic or multiphase food matrices further prevent phase separation and oil loss [44]. Moreover, encapsulation can mask or modulate intense citrus flavor notes, enhancing consumer acceptance [45], while reducing the effective dose required to achieve functional effects, thereby limiting costs and minimizing sensory off-notes [46]. By forming a physical barrier between the volatile oil core and the external environment, encapsulation enables targeted and sustained delivery of LEO, which is particularly advantageous in lipid-rich products such as margarine, dairy alternatives, and emulsified sauces [47].

### 5.2. Encapsulation Materials and Carriers

A wide range of wall materials has been investigated for LEO encapsulation, selected according to functional performance, target food matrices, regulatory acceptance, and economic feasibility.

Biopolymers. Polysaccharides such as pectin, alginate, chitosan, and gum arabic are widely used due to their biodegradability, film-forming ability, and compatibility with food systems. Protein-based carriers, including gelatin and whey protein isolate, offer additional emulsifying capacity and enhanced barrier properties [48].

Lipid-based carriers. Lipid materials such as waxes, triglycerides, and phospholipids, forming liposomal structures, are particularly suitable for encapsulating lipophilic compounds like LEO and can modulate release behavior during storage and gastrointestinal digestion [49].

Inorganic materials. Emerging strategies employ amorphous or mesoporous silica particles to entrap LEO, providing high encapsulation efficiency and reduced volatilization through the formation of rigid protective shells. These systems offer excellent thermal and oxidative stability, although regulatory considerations remain critical [50,51].

The selection of wall materials strongly depends on physicochemical compatibility with LEO, the intended delivery mechanism, regulatory constraints, and cost considerations [52].

### 5.3. Encapsulation Techniques

Encapsulation techniques optimized for LEO differ substantially in terms of particle size, encapsulation efficiency, scalability, and release behavior (Table 3).

Among these approaches, spray drying remains the most widely implemented at industrial scale, commonly employing maltodextrin and gum arabic to form protective protein–polysaccharide matrices [60]. Complex coacervation relies on electrostatic interactions between oppositely charged biopolymers (e.g., gelatin–gum arabic), generating dense shells that effectively limit volatile losses [61].

**Figure 2:**
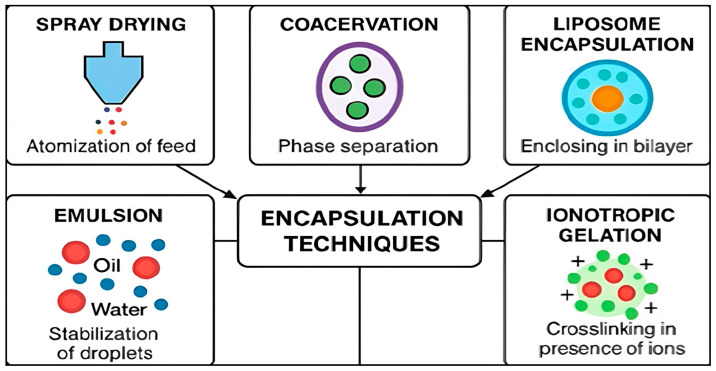
Schematic representation of key encapsulation techniques for lemon essential oil and their structural architectures.

Sol–gel encapsulation enables the formation of silica shells under mild conditions, providing superior thermal and oxidative protection for highly volatile LEO constituents [62]. Nanoemulsions, typically produced via ultrasonication or high-pressure homogenization, enhance aqueous dispersibility and bioavailability of LEO [63,64]. Liposomal systems encapsulate LEO within phospholipid bilayers, enabling digestion-triggered release and suitability for functional foods and nutraceutical applications [65] (Figure 2). 

### 5.4. Mechanisms of Protection and Release

From a mechanistic perspective, essential oil encapsulation and release can be rationalized according to three main mechanisms: physical embedding, chemical inclusion, and physicochemical adsorption. Encapsulation protects LEO by physically entrapping volatile compounds and forming effective barriers against environmental stressors such as oxygen, moisture, light, and heat [66]. By limiting diffusion and reducing exposure to pro-oxidant conditions, encapsulation minimizes volatilization and oxidative degradation during processing and storage [67]. Depending on the encapsulation strategy, protection and release of LEO are governed by different underlying mechanisms, including physical embedding, chemical inclusion, and physicochemical adsorption, each involving specific interaction forces between the oil and the carrier matrix [68].

The release kinetics of encapsulated LEO strongly depend on the nature of the carrier matrix and on environmental conditions, including pH, temperature, and enzymatic activity. In physical embedding systems, such as spray-dried powders, nanoemulsions, and complex coacervates, LEO droplets are mechanically entrapped within a continuous matrix, and release occurs through matrix swelling, erosion, or rupture [69]. In aqueous emulsions, an immediate release is often observed as carrier shells rapidly swell or rupture, contributing to flavor impact or antimicrobial action [70]. This rapid release behavior is particularly associated with nanoemulsified systems and high interfacial surface area. In contrast, sustained release is characteristic of solid matrices such as spray-dried powders or coacervates, enabling prolonged bioactivity during storage [71]. Dense solid carriers limit molecular diffusion and enhance oxidative stability by reducing oxygen permeability [72]. Furthermore, chemical inclusion systems, including liposomal and inorganic carriers, rely on specific interactions such as hydrogen bonding, van der Waals forces, or coordination interactions, resulting in slower and more controlled release profiles [73]. Furthermore, triggered release may occur in response to pH variations, enzymatic digestion, or temperature changes, allowing targeted delivery of LEO within food systems or along the gastrointestinal tract [74]. In adsorptive systems, physicochemical interactions such as hydrophobic and electrostatic forces play a key role in modulating release behavior, which is highly sensitive to changes in pH, moisture, and temperature [75,76]. Overall, the release kinetics of encapsulated LEO are determined by the combined effects of encapsulation mechanism, interaction forces, carrier composition, and external stimuli, enabling targeted and functional delivery in complex food matrices [77].

### 5.5. Performance in Food Systems

Encapsulation substantially enhances the stability and functional efficacy of LEO in real food applications. In terms of oxidative stability, LEO encapsulated in amorphous silica significantly reduced peroxide and TBARS values in margarine, thereby extending shelf life through improved control of lipid oxidation [51]. In the context of active packaging, pectin-based films incorporating LEO microcapsules exhibited controlled antioxidant release and antimicrobial activity, highlighting their potential for food coating and packaging applications [78]. In dairy and emulsion-based systems, nanoemulsified LEO improved antioxidant performance without adversely affecting sensory properties in cream and cheese models [79]. Moreover, spray-dried LEO powders stabilized with whey protein and gum arabic effectively preserved volatile compounds during baking and storage, resulting in enhanced sensory quality [80]. Collectively, these findings clearly illustrate the ability of encapsulation technologies to improve both shelf life and functional performance of LEO in complex food matrices.

### 5.6. Challenges and Future Considerations

Despite the demonstrated benefits, several challenges must be addressed to enable widespread industrial adoption of LEO encapsulation:

- achieving high encapsulation efficiency while minimizing oil loss during processing remains critical for commercial viability [81];
- preventing premature leakage and volatilization during long-term storage, particularly under humid or oxygen-rich conditions, remains technically demanding [66];
- regulatory and safety aspects associated with nanoencapsulation materials require careful evaluation to ensure compliance with food-grade standards [82];
- balancing cost-effectiveness with scalability is essential for industrial implementation [83].

Emerging hybrid systems, including Pickering emulsions stabilized by food-grade particles and layer-by-layer (LbL) assembled microcapsules, are actively being explored to improve encapsulation efficiency, stability, and release control, showing promising potential for advanced LEO-based food applications [51].

## 6. Application in Margarine and Lipid-Based Matrices

Although many encapsulation systems and wall materials are initially developed in aqueous or multiphase environments, their application to lipid-based foods requires careful consideration of interfacial behavior, oxygen diffusion, and matrix compatibility. These aspects are critically discussed in the context of margarine systems in this review. Light margarines are particularly susceptible to lipid oxidation due to their high content of polyunsaturated fatty acids (PUFAs) and generally limited intrinsic antioxidant protection. This vulnerability often results in oxidative rancidity, off-flavor development, and reduced shelf life, representing a major challenge for product stability and consumer acceptance. Within this framework, light margarine is not presented as an isolated case study, but rather as a model lipid-based system that enables comparison of encapsulation strategies under conditions of high oxidative stress. Accordingly, the incorporation of lemon essential oil (LEO), rich in bioactive compounds such as limonene, has been extensively investigated as a natural strategy to counteract oxidative degradation in margarine formulations [18,84].

### 6.1. Enhancement of Oxidative Stability and Shelf Life

Multiple studies have demonstrated that encapsulated LEO significantly enhances the oxidative stability of margarine when compared with control formulations lacking antioxidants or containing free (non-encapsulated) LEO. In particular, encapsulated LEO has been reported to reduce primary and secondary oxidation markers by 25–60%, depending on the encapsulation system and storage conditions, highlighting its effectiveness as a natural antioxidant strategy. Encapsulation systems such as biopolymer-based microencapsulation effectively protect volatile and oxidation-sensitive LEO constituents during processing and storage, thereby limiting degradation and volatilization losses [85].

For instance, Himed et al. [18] reported that margarine enriched with silica-encapsulated LEO exhibited a reduction in peroxide value (PV) of approximately 40–50% after 60 days of refrigerated storage, compared with both free-LEO-enriched and untreated control samples. Similarly, thiobarbituric acid reactive substances (TBARS) values were reduced by nearly 35–45%, indicating a substantial inhibition of secondary lipid oxidation processes. This protective effect translated into a delay of lipid oxidation by at least 2–3 weeks, ultimately extending shelf life while preserving the physicochemical and sensory attributes of the margarine. Comparable improvements in oxidative stability have also been reported for biopolymer-based encapsulation systems, with encapsulated LEO maintaining acceptable oxidation levels throughout storage periods exceeding 90 days, whereas control samples showed rapid deterioration after 30–45 days [51].

### 6.2. Controlled Release and Sensory Quality Preservation

Encapsulation enables controlled release of LEO constituents, allowing the use of lower essential oil concentrations without compromising functional efficacy. Reduced dosages minimize the risk of excessive citrus aroma or off-notes, which could otherwise negatively affect sensory acceptance of margarine products [85].

Biopolymer-based carriers such as pectin and chitosan have proven particularly effective in providing sustained and controlled release of LEO within lipid matrices during storage. This controlled diffusion maintains antioxidant activity over time while preserving a balanced flavor profile [51,86]. In addition, these natural polymers offer advantages in terms of biodegradability, regulatory acceptance, and compatibility with food processing requirements.

### 6.3. Optimization of Encapsulation Materials

The optimization of encapsulating materials plays a critical role in maximizing LEO retention and functional performance in margarine systems. Synergistic combinations of wall materials have been shown to significantly enhance encapsulation efficiency and volatile retention. For example, ternary blends of gum arabic, maltodextrin, and modified starch, formulated at optimized ratios, achieved encapsulation efficiencies approaching 78% and improved limonene retention in spray-dried powders intended for antioxidant or flavor delivery [85]. Such optimized carrier systems contribute to improved emulsification, film-forming capacity, and microcapsule structural stability, which are essential for reliable performance in lipid-based matrices.

In addition to wall material composition, encapsulation efficiency and loading capacity are strongly influenced by formulation and processing parameters, including wall-to-core ratio, solid content, molecular characteristics of the carrier materials, emulsification efficiency, and drying conditions [60]. The wall-to-core ratio is one of the most critical parameters governing active ingredient loading and encapsulation efficiency. Increasing this ratio generally enhances oil retention by ensuring complete interfacial coverage of oil droplets and reducing surface oil; however, excessively high ratios may dilute the active compound concentration and reduce delivery efficiency [87]. Optimal wall-to-core ratios for essential oil encapsulation by spray drying are typically reported between 3:1 and 5:1, offering a balance between high encapsulation efficiency and acceptable payload levels. For example, in the spray-drying of sea buckthorn berry oil, higher carrier-to-oil ratios (4:1) were associated with improved encapsulation efficiency and powder properties, suggesting that increased wall material relative to core enhances encapsulation performance [88].

The physicochemical properties of encapsulation materials, such as molecular weight, polarity, and glass transition temperature, play a decisive role in volatile retention. Wall materials with higher glass transition temperatures form rigid, glassy matrices after drying, limiting molecular mobility and reducing diffusion-driven losses of volatile compounds during storage, whereas low-Tg materials may increase permeability and promote premature release [89].

Emulsification-related factors, including emulsifier type, homogenization pressure, and droplet size distribution, also critically affect encapsulation performance. Smaller and more uniform droplets provide a larger interfacial area for wall material adsorption, resulting in more homogeneous microcapsules, improved film continuity, and higher encapsulation efficiency [90].

Processing parameters, particularly spray-drying inlet temperature, outlet temperature, and drying rate, further influence microcapsule morphology and volatile retention. Rapid crust formation at optimized inlet temperatures favors efficient entrapment of essential oils, while excessive thermal stress accelerates volatilization and oxidative degradation of sensitive compounds [60].

Overall, the encapsulation efficiency and functional performance of LEO-loaded systems are governed by the combined effects of wall material composition, formulation ratios, interfacial properties, and processing conditions. Rational optimization of these parameters is therefore essential to achieve high loading capacity, improved volatile retention, and long-term stability of active ingredients in margarine and other lipid-based food products [91].

### 6.4. Impact on Product Acceptability and Regulatory Aspects

Beyond oxidative stabilization, the incorporation of encapsulated LEO has been reported to exert minimal impact on the physicochemical properties of light margarine, maintaining texture, appearance, and spreadability comparable to conventional formulations [18]. Sensory evaluations further indicate that appropriately dosed encapsulated LEO can enhance overall product acceptability by balancing flavor intensity with effective antioxidant protection.

From a regulatory perspective, many biopolymer carriers employed for LEO encapsulation, such as pectin, gum arabic, and chitosan, as well as silica-based materials, are classified as Generally Recognized as Safe (GRAS) or are approved food additives, enabling their application in edible lipid matrices. Nevertheless, the use of micro- and nano-scale delivery systems may require case-by-case safety assessment depending on regulatory jurisdiction.

### 6.5. Industrial and Commercial Applications of LEO Encapsulation

Encapsulation strategies for lemon essential oil (LEO) have been increasingly explored for industrial-scale applications in lipid-based food systems, with scalable techniques such as spray-drying, nanoemulsions, and microencapsulation being widely investigated for their capacity to produce stable encapsulates at high throughput [92,93].

Spray-drying is one of the most industrially established encapsulation methods, enabling the conversion of LEO into dry microcapsules with improved handling, storage stability, and controlled release properties [60]. Nanoemulsion-based approaches and liposomal systems have also shown promise for large-scale production, offering advantages in transparency, droplet size uniformity, and enhanced bioavailability of active compounds in aqueous food matrices [94].

**Figure 3:**
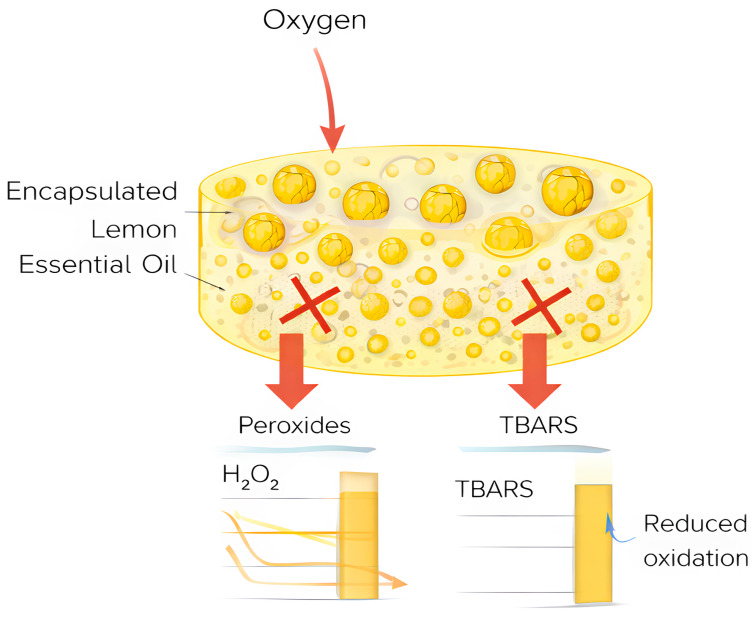
Schematic representation of the incorporation of encapsulated lemon essential oil into margarine and its role in oxidative stability control.

In commercial food applications, LEO-encapsulated formulations have been successfully incorporated into margarines, spreads, and other lipid-rich products, where they provide antioxidant protection and may contribute to improved shelf life without adversely affecting sensory quality [95] (Figure 3). 

Microencapsulation facilitates standardized dosing and mitigates volatilization and oxidation of flavor compounds during processing, storage, and distribution, which are major concerns for essential oils in food systems [96].

Commercial implementation of these encapsulation technologies requires careful consideration of material costs, process efficiency, and regulatory compliance. Common biopolymer carriers such as gum arabic, pectin, and chitosan are classified as generally recognized as safe (GRAS) or approved food additives, facilitating their use in industrial food applications [83,96]. Nevertheless, optimization of wall-to-core ratios, drying parameters, and emulsification conditions remains essential to achieve consistent encapsulation efficiency, high payload, and reproducible functional performance at scale [81,91].

## 7. Safety and Regulatory Considerations

In parallel with technological advances, safety and regulatory compliance remain critical aspects for the successful application of encapsulated lemon essential oil (LEO) in food systems. Recent developments in encapsulation technologies, including nanoemulsions, liposomes, and biodegradable polymer-based carriers, have significantly enhanced the stability, controlled release, and bioavailability of LEO, thereby broadening its potential applications in food preservation, pharmaceuticals, and cosmetics [92,97,98,99].

However, these innovations also raise important safety considerations, particularly when nano- and micro-scale delivery systems are involved. Nanoencapsulation may alter the biological interactions, absorption, and metabolic fate of essential oil constituents compared with their non-encapsulated counterparts, necessitating thorough toxicological evaluation and risk assessment [73,92].

From a regulatory perspective, the use of encapsulated LEO must comply with existing food additive and flavoring regulations. In the European Union, relevant frameworks include Regulation (EC) No. 1333/2008 on food additives, while in the United States, the Generally Recognized as Safe (GRAS) status and FEMA guidelines govern the use of flavoring substances. Although many carrier materials employed for encapsulation, such as pectin, gum arabic, chitosan, and food-grade silica, are already approved for food use, the regulatory status of novel encapsulation systems may require case-by-case evaluation. Consequently, harmonization of international regulatory frameworks remains essential to facilitate safe commercialization while ensuring consumer protection.

## 8. Future Trends and Perspectives

Future research on lemon essential oil (LEO) encapsulation is expected to increasingly focus on the development of smart delivery systems capable of responding dynamically to environmental stimuli such as pH variations, temperature changes, or enzymatic activity. These responsive systems could enable targeted release and improved functional efficiency, optimizing the antioxidant and antimicrobial effects of LEO while minimizing losses and undesired interactions.

Another promising research direction involves the incorporation of encapsulated LEO into active and intelligent packaging materials. Embedding micro- or nanoencapsulated LEO within biodegradable polymer matrices may provide prolonged antimicrobial and antioxidant activity at the food–packaging interface, thereby extending shelf life and reducing reliance on synthetic preservatives.

Sustainability will continue to act as a major driver for innovation, promoting the use of edible, biodegradable, and renewable encapsulation materials. Such approaches are aligned with clean-label trends and address growing concerns related to environmental impact and plastic waste.

In parallel, comprehensive life cycle assessment (LCA) and techno-economic analyses are required to evaluate the scalability, environmental footprint, and cost-effectiveness of encapsulation technologies. These evaluations are crucial for guiding industrial implementation and supporting the transition from laboratory-scale systems to commercially viable solutions.

Finally, expanding the application of encapsulated LEO to a broader range of food products, including emulsion-based systems, dairy alternatives, and bakery goods, offers considerable potential. Encapsulation can effectively mitigate stability and sensory challenges, enabling the integration of LEO’s functional properties into diverse food matrices while preserving overall product quality.

Collectively, these perspectives underscore the importance of a multidisciplinary approach that integrates materials science, food technology, toxicology, and sustainability to advance the practical application of LEO-based encapsulation systems.

## 9. Conclusions

Encapsulation markedly enhances the functional performance of lemon essential oil in food applications by improving its physicochemical stability, enabling controlled release, and increasing compatibility with complex food matrices. These advantages allow encapsulated LEO to function as an effective natural alternative to synthetic preservatives, in line with consumer demand for clean-label and minimally processed foods. Continued advances in encapsulation technologies, together with the development of biodegradable and food-grade carrier materials, are expected to further improve the efficacy and safety of LEO-based formulations. Moreover, integrating encapsulation strategies with sustainability and regulatory considerations will be pivotal for accelerating industrial adoption. Ultimately, ongoing interdisciplinary research and development will be essential to fully exploit the potential of lemon essential oil as a multifunctional ingredient in next-generation food technologies.

## Figures and Tables

**Table 1:** Chemical Composition and Biological Activities of Lemon Essential Oil (LEO).

Compound Class/Major Constituent	Typical Content in LEO	Associated Biological Activity	Main Mechanism of Action	References
Monoterpenes (major fraction)	≈85–95%	Antioxidant	Scavenging of reactive oxygen species (ROS), hydrogen atom donation, inhibition of lipid peroxidation	[3,17,18,19]
Limonene	55.4–66.75%	Antioxidant, moderate antimicrobial	Free radical neutralization, alteration of microbial cell membranes	[3,18,19,20]
γ-Terpinene	3.10–10.3%	Antioxidant	Strong radical-scavenging activity, synergistic effects with limonene	[3,18,19,21]
β-Pinene	12.6–13.92%	Antimicrobial	Disruption of bacterial membrane integrity	[3,18,19,22]
Citral (geranial + neral)	3–10.39%	Strong antimicrobial	Membrane disorganization, intracellular enzyme inhibition	[19,23]
Linalool	0.5%	Antimicrobial, synergistic	Increased membrane permeability, synergistic interactions	[19]
α-Terpineol	0.7%	Antioxidant, antimicrobial	Interaction with membrane lipids and cellular enzymes (ATPase) disruption	[19]
Minor compounds (aldehydes, ketones, esters)	2–5%	Sensory and biological modulation	Additive and synergistic effects contributing to overall bioactivity	[3,18,19,23]
Overall chemical profile (chemotype)	Variable	Global biological efficacy	Influenced by cultivar, geographical origin, maturity stage, extraction method, and storage conditions	[24]

**Table 2:** Comparative overview of extraction techniques for lemon essential oil.

Method	Temperature Range	Extraction Time	Yield Efficiency	Thermal Degradation Risk
Steam distillation	100–110 °C	1–3 h	Medium	High
Cold pressing	Ambient	Fast	Low–Medium	Low
MAHD	60–90 °C	30–60 min	High	Low
UAE	<50 °C	15–45 min	Medium–High	Very low
Supercritical CO_2_	35–50 °C (200–300 bar)	1–2 h	High	Very low

**Table 3:** Main encapsulation techniques applied to lemon essential oil.

Technique	Mechanism	Particle Size	Encapsulation Efficiency	Release Profile	Remarks	References
Spray drying	Atomization and dehydration of emulsion	11.9–44.4 µm	5.7–97%	Fast or sustained	Industrially scalable; commonly used with maltodextrin, gum arabic; cost-effective	[53,54]
Complex coacervation	Phase separation in colloidal systems	81.63–156.74 µm	95%	Moderate to sustained	Precise shell control; examples: gelatin–gum arabic, whey protein–pectin; good for heat-sensitive compounds	[42,55,56]
Sol–gel encapsulation	Silica polymerization around droplets	0.1–10 µm	>85%	Slow, temperature-stable	Suitable for heat-sensitive oils; can combine with organosilicates; high oxidative protection	[51]
Nanoemulsification	High-shear or ultrasonic dispersion	80–125 nm	88–96%	Rapid	Improved bioavailability; can use Tween 80, lecithin, or protein emulsifiers; suitable for beverages	[57,58]
Liposomal entrapment	Phospholipid bilayer vesicles	110–117 nm	91–94%	Slow, digestion-triggered	Suitable for dairy and nutraceuticals; examples: soy lecithin, egg lecithin; enhanced cellular uptake	[59]

## Data Availability

No new data was created in this study. Data sharing is not applicable to this article.
